# 2-Chloro-*N*-phenyl­acetamide

**DOI:** 10.1107/S160053680801266X

**Published:** 2008-05-03

**Authors:** B. Thimme Gowda, Jozef Kožíšek, Miroslav Tokarčík, Hartmut Fuess

**Affiliations:** a Department of Chemistry, Mangalore University, Mangalagangotri 574 199, Mangalore, India; b Faculty of Chemical and Food Technology, Slovak Technical University, Radlinského 9, SK-812 37 Bratislava, Slovak Republic; c Institute of Materials Science, Darmstadt University of Technology, Petersenstrasse 23, D-64287 Darmstadt, Germany

## Abstract

In the title compound, C_8_H_8_ClNO, the conformations of the N—H and C=O bonds are *anti* to each other, but the C—Cl and C=O bonds in the side chain are *syn*. The mol­ecules are linked by N—H⋯O hydrogen bonds into infinite chains running in the [101] direction.

## Related literature

For the synthesis, see: Gowda *et al.* (2003 ▶). For related structures, see: Gowda *et al.* (2007 ▶, 2008 ▶).
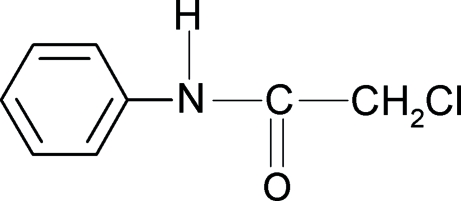


## Experimental

### 

#### Crystal data

C_8_H_8_ClNO
*M* *_r_* = 169.6
Monoclinic, 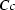

*a* = 5.0623 (15) Å
*b* = 18.361 (6) Å
*c* = 9.115 (2) Å
β = 102.13 (3)°
*V* = 828.3 (4) Å^3^
*Z* = 4
Mo *K*α radiation
μ = 0.40 mm^−1^
*T* = 297 (2) K
0.41 × 0.24 × 0.17 mm

#### Data collection

Oxford Diffraction Xcalibur diffractometer
Absorption correction: analytical [*CrysAlis RED* (Oxford Diffraction, 2006 ▶), using a multifaceted crystal model based on expressions derived by Clark & Reid (1995 ▶)] *T*_min_ = 0.905, *T*_max_ = 0.938
2388 measured reflections
1067 independent reflections
385 reflections with *I* > 2σ(*I*)
*R*_int_ = 0.046

#### Refinement

*R*[*F* ^2^ > 2σ(*F* ^2^)] = 0.036
*wR*(*F* ^2^) = 0.086
*S* = 0.96
1067 reflections
106 parameters
2 restraints
H-atom parameters constrained
Δρ_max_ = 0.1 e Å^−3^
Δρ_min_ = −0.11 e Å^−3^
Absolute structure: Flack (1983 ▶), 254 Friedel pairs
Flack parameter: 0.04 (11)

### 

Data collection: *CrysAlis CCD* (Oxford Diffraction, 2006 ▶); cell refinement: *CrysAlis RED* (Oxford Diffraction, 2006 ▶); data reduction: *CrysAlis RED*; program(s) used to solve structure: *SHELXS97* (Sheldrick, 2008 ▶); program(s) used to refine structure: *SHELXL97* (Sheldrick, 2008 ▶); molecular graphics: *ORTEP-3* (Farrugia, 1997 ▶) and *DIAMOND* (Brandenburg, 2002 ▶); software used to prepare material for publication: *SHELXL97*, *PLATON* (Spek, 2003 ▶) and *WinGX* (Farrugia, 1999 ▶).

## Supplementary Material

Crystal structure: contains datablocks I, global. DOI: 10.1107/S160053680801266X/hb2728sup1.cif

Structure factors: contains datablocks I. DOI: 10.1107/S160053680801266X/hb2728Isup2.hkl

Additional supplementary materials:  crystallographic information; 3D view; checkCIF report

## Figures and Tables

**Table 1:** Hydrogen-bond geometry (Å, °)

*D*—H⋯*A*	*D*—H	H⋯*A*	*D*⋯*A*	*D*—H⋯*A*
N1—H1N⋯O1^i^	0.86	2.05	2.848 (5)	155

Symmetry code: (i) 
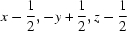
.

**Fig. 1.**
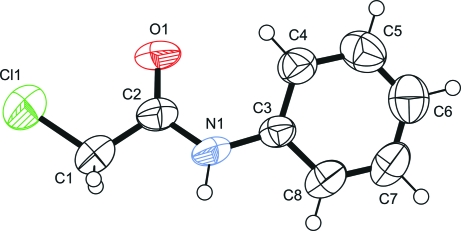
Molecular structure of (I) with displacement ellipsoids for the non-hydrogen atoms drawn at the 50% probability level.

**Fig. 2.**
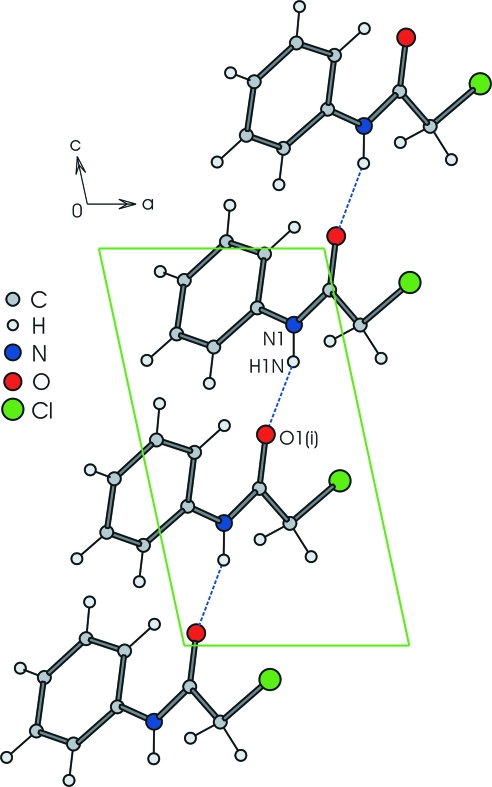
Part of the packing for (I) viewed down the b axis showing the chains arising from N-H···O hydrogen bonds Symmetry code (i): x - 1/2,-y + 1/2,z - 1/2.

## supplementary crystallographic information

### Comment

In the present work, the structure of the title compoud, (I), 2-chloro-*N*-(phenyl)-acetamide (NPCA) has been determined, as part of a study of the effect of ring and side chain substitutions on the solid state geometry of aromatic amides (Gowda *et al.*, 2007; 2008). The conformations of the N—H and C=O bonds are *anti* to each other, but the C—Cl and C=O bonds in the side chain are *syn* to each other (Fig. 1), similar to that observed in 2-chloro-*N*-(2-chlorophenyl)-acetamide (Gowda *et al.*, 2007)and 2-chloro-*N*-(3-methylphenyl)-acetamide (Gowda *et al.*, 2008) with similar bond parameters. Further, the amide group –NHCO– in (I) makes a dihedral angle of 16.0 (8)° with the phenyl ring.

Part of the packing for (I) viewed down the *b* axis is shown in Fig. 2. Infinite chains running along the base vector [101] are formed by N-H···O hydrogen bonds (Table 1).

### Experimental

The title compound was prepared according to the literature method (Gowda *et al.*, 2003) and colourless prisms of (I) were recrystallised from an ethanol solution.

### Refinement

The H atoms were placed in calculated positions (C-H = 0.93Å, N-H = 0.86Å) and refined as riding with *U*_iso_(H) = 1.2*U*_eq_(C,*N*).

### Crystal data

**Table:**

C_8_H_8_ClNO	*F*_000_ = 352
*M**_r_* = 169.6	*D*_x_ = 1.36 Mg m^−^^3^
Monoclinic, *C**c*	Mo *K*α radiation λ = 0.71073 Å
Hall symbol: C -2yc	Cell parameters from 159 reflections
*a* = 5.0623 (15) Å	θ = 4.9–25.1º
*b* = 18.361 (6) Å	µ = 0.40 mm^−^^1^
*c* = 9.115 (2) Å	*T* = 297 (2) K
β = 102.13 (3)º	Prism, colorless
*V* = 828.3 (4) Å^3^	0.41 × 0.24 × 0.17 mm
*Z* = 4	

### Data collection

**Table:**

Oxford Diffraction Xcalibur System diffractometer	1067 independent reflections
Radiation source: Enhance (Mo) X-ray Source	385 reflections with *I* > 2σ(*I*)
Monochromator: graphite	*R*_int_ = 0.046
Detector resolution: 10.4340 pixels mm^-1^	θ_max_ = 26º
*T* = 297(2) K	θ_min_ = 4.3º
ω scans	*h* = −6→6
Absorption correction: analytical[CrysAlis RED (Oxford Diffraction, 2006), using a multifaceted crystal model based on expressions derived by Clark & Reid (1995)]	*k* = −22→22
*T*_min_ = 0.905, *T*_max_ = 0.938	*l* = −9→11
2388 measured reflections	

### Refinement

**Table:**

Refinement on *F*^2^	Hydrogen site location: inferred from neighbouring sites
Least-squares matrix: full	H-atom parameters constrained
*R*[*F*^2^ > 2σ(*F*^2^)] = 0.036	[exp(3.70(sinθ/λ)^2^)]/[σ^2^(*F*_o_^2^) + (0.035*P*)^2^] where *P* = 0.33333*F*_o_^2^ + 0.66667*F*_c_^2^
*wR*(*F*^2^) = 0.086	(Δ/σ)_max_ < 0.001
*S* = 0.96	Δρ_max_ = 0.1 e Å^−^^3^
1067 reflections	Δρ_min_ = −0.11 e Å^−^^3^
106 parameters	Extinction correction: none
2 restraints	Absolute structure: Flack (1983), 254 Friedel pairs
Primary atom site location: structure-invariant direct methods	Flack parameter: 0.04 (11)
Secondary atom site location: difference Fourier map	

### Special details

**Table:**

Geometry. All e.s.d.'s (except the e.s.d. in the dihedral angle between two l.s. planes) are estimated using the full covariance matrix. The cell e.s.d.'s are taken into account individually in the estimation of e.s.d.'s in distances, angles and torsion angles; correlations between e.s.d.'s in cell parameters are only used when they are defined by crystal symmetry. An approximate (isotropic) treatment of cell e.s.d.'s is used for estimating e.s.d.'s involving l.s. planes.
Refinement. Refinement of *F*^2^ against ALL reflections. The weighted *R*-factor *wR* and goodness of fit *S* are based on *F*^2^, conventional *R*-factors *R* are based on *F*, with *F* set to zero for negative *F*^2^. The threshold expression of *F*^2^ > σ(*F*^2^) is used only for calculating *R*-factors(gt) *etc*. and is not relevant to the choice of reflections for refinement. *R*-factors based on *F*^2^ are statistically about twice as large as those based on *F*, and *R*- factors based on ALL data will be even larger.

### Fractional atomic coordinates and isotropic or equivalent isotropic displacement parameters (Å^2^)

**Table:**

	*x*	*y*	*z*	*U*_iso_*/*U*_eq_	
Cl1	1.3492 (4)	0.15000 (9)	0.9151 (2)	0.1082 (7)	
C1	1.0871 (11)	0.1998 (3)	0.8076 (6)	0.0824 (18)	
H1A	1.1488	0.2215	0.7237	0.099*	
H1B	0.939	0.1671	0.7672	0.099*	
C2	0.9849 (11)	0.2595 (3)	0.8961 (6)	0.0629 (17)	
N1	0.7939 (8)	0.3008 (2)	0.8086 (4)	0.0642 (13)	
H1N	0.7565	0.2897	0.7149	0.077*	
O1	1.0653 (7)	0.26846 (19)	1.0314 (3)	0.0833 (13)	
C3	0.6481 (10)	0.3597 (3)	0.8507 (6)	0.0517 (13)	
C4	0.7302 (11)	0.3975 (3)	0.9862 (6)	0.0670 (17)	
H4	0.888	0.3845	1.0536	0.08*	
C5	0.5727 (15)	0.4542 (3)	1.0177 (7)	0.082 (2)	
H5	0.6222	0.478	1.1094	0.099*	
C6	0.3490 (16)	0.4762 (3)	0.9197 (10)	0.0824 (18)	
H6	0.2513	0.516	0.9428	0.099*	
C7	0.2640 (13)	0.4399 (4)	0.7850 (7)	0.082 (2)	
H7	0.1068	0.4538	0.7183	0.099*	
C8	0.4153 (10)	0.3835 (4)	0.7525 (6)	0.0676 (16)	
H8	0.3614	0.3598	0.6609	0.081*	

### Atomic displacement parameters (Å^2^)

**Table:**

	*U*^11^	*U*^22^	*U*^33^	*U*^12^	*U*^13^	*U*^23^
Cl1	0.1120 (13)	0.1207 (13)	0.0829 (10)	0.0402 (13)	−0.0004 (9)	0.0083 (13)
C1	0.079 (4)	0.091 (4)	0.067 (4)	0.022 (4)	−0.006 (3)	0.006 (4)
C2	0.070 (4)	0.071 (4)	0.045 (3)	−0.003 (3)	0.005 (3)	0.004 (4)
N1	0.066 (3)	0.084 (3)	0.035 (3)	0.012 (3)	−0.006 (2)	0.003 (3)
O1	0.102 (3)	0.097 (3)	0.039 (2)	0.010 (2)	−0.013 (2)	−0.005 (2)
C3	0.051 (4)	0.062 (4)	0.041 (3)	−0.001 (3)	0.008 (3)	0.002 (3)
C4	0.055 (4)	0.084 (4)	0.059 (4)	0.005 (4)	0.007 (3)	−0.004 (3)
C5	0.083 (5)	0.097 (5)	0.073 (5)	−0.005 (5)	0.029 (4)	−0.014 (4)
C6	0.078 (5)	0.071 (4)	0.101 (5)	0.002 (5)	0.027 (4)	0.005 (5)
C7	0.066 (5)	0.094 (5)	0.081 (5)	0.019 (5)	0.005 (4)	0.020 (5)
C8	0.053 (4)	0.090 (5)	0.058 (4)	0.003 (3)	0.006 (3)	0.014 (3)

### Geometric parameters (Å, °)

**Table:**

Cl1—C1	1.735 (5)	C4—C5	1.378 (7)
C1—C2	1.515 (6)	C4—H4	0.93
C1—H1A	0.97	C5—C6	1.349 (8)
C1—H1B	0.97	C5—H5	0.93
C2—O1	1.226 (6)	C6—C7	1.384 (9)
C2—N1	1.350 (6)	C6—H6	0.93
N1—C3	1.407 (6)	C7—C8	1.357 (7)
N1—H1N	0.86	C7—H7	0.93
C3—C8	1.392 (6)	C8—H8	0.93
C3—C4	1.401 (7)		
			
C2—C1—Cl1	112.8 (4)	C5—C4—C3	118.7 (6)
C2—C1—H1A	109	C5—C4—H4	120.7
Cl1—C1—H1A	109	C3—C4—H4	120.7
C2—C1—H1B	109	C6—C5—C4	122.0 (6)
Cl1—C1—H1B	109	C6—C5—H5	119
H1A—C1—H1B	107.8	C4—C5—H5	119
O1—C2—N1	124.3 (6)	C5—C6—C7	120.3 (6)
O1—C2—C1	123.7 (6)	C5—C6—H6	119.8
N1—C2—C1	112.0 (5)	C7—C6—H6	119.8
C2—N1—C3	128.5 (5)	C8—C7—C6	118.5 (6)
C2—N1—H1N	115.7	C8—C7—H7	120.8
C3—N1—H1N	115.7	C6—C7—H7	120.8
C8—C3—C4	117.8 (5)	C7—C8—C3	122.6 (6)
C8—C3—N1	119.2 (5)	C7—C8—H8	118.7
C4—C3—N1	123.0 (5)	C3—C8—H8	118.7
			
Cl1—C1—C2—O1	−4.8 (7)	N1—C3—C4—C5	−179.6 (5)
Cl1—C1—C2—N1	175.8 (4)	C3—C4—C5—C6	−2.8 (9)
O1—C2—N1—C3	−1.1 (9)	C4—C5—C6—C7	2.7 (9)
C1—C2—N1—C3	178.3 (5)	C5—C6—C7—C8	−2.0 (9)
C2—N1—C3—C8	−164.2 (5)	C6—C7—C8—C3	1.7 (8)
C2—N1—C3—C4	17.8 (8)	C4—C3—C8—C7	−1.8 (8)
C8—C3—C4—C5	2.3 (8)	N1—C3—C8—C7	−180.0 (5)

### Hydrogen-bond geometry (Å, °)

**Table:**

*D*—H···*A*	*D*—H	H···*A*	*D*···*A*	*D*—H···*A*
N1—H1N···O1^i^	0.86	2.05	2.848 (5)	155

Symmetry codes: (i) *x*−1/2, −*y*+1/2, *z*−1/2.
